# Targeting anti-apoptotic Bcl-2 by AT-101 to increase radiation efficacy: data from in vitro and clinical pharmacokinetic studies in head and neck cancer

**DOI:** 10.1186/s13014-015-0474-9

**Published:** 2015-07-30

**Authors:** Shuraila F. Zerp, T. Rianne Stoter, Frank J. P. Hoebers, Michiel W. M. van den Brekel, Ria Dubbelman, Gitta K. Kuipers, M. Vincent M. Lafleur, Ben J. Slotman, Marcel Verheij

**Affiliations:** Department of Biological Stress Response, The Netherlands Cancer Institute, Amsterdam, The Netherlands; Department of Radiation Oncology, VU University Medical Center, Amsterdam, The Netherlands; Department of Radiation Oncology (MAASTRO), GROW - School for Oncology and Developmental Biology, Maastricht University Medical Centre, Maastricht, The Netherlands; Department of Head and Neck Surgery and Oncology, The Netherlands Cancer Institute, Amsterdam, The Netherlands; Department of Radiotherapy, The Netherlands Cancer Institute, Amsterdam, The Netherlands; The Netherlands Cancer Institute, Plesmanlaan 121, 1066 CX Amsterdam, The Netherlands

**Keywords:** Radiation, Head and neck cancer, Apoptosis, Bcl-2

## Abstract

**Background:**

Pro-survival Bcl-2 family members can promote cancer development and contribute to treatment resistance. Head and neck squamous cell carcinoma (HNSCC) is frequently characterized by overexpression of anti-apoptotic Bcl-2 family members. Increased levels of these anti-apoptotic proteins have been associated with radio- and chemoresistance and poor clinical outcome. Inhibition of anti-apoptotic Bcl-2 family members therefore represents an appealing strategy to overcome resistance to anti-cancer therapies. The aim of this study was to evaluate combined effects of radiation and the pan-Bcl-2 inhibitor AT-101 in HNSCC *in vitro*. In addition, we determined human plasma levels of AT-101 obtained from a phase I/II trial, and compared these with the effective *in vitro* concentrations to substantiate therapeutic opportunities.

**Methods:**

We examined the effect of AT-101, radiation and the combination on apoptosis induction and clonogenic survival in two HNSCC cell lines that express the target proteins. Apoptosis was assessed by *bis*-benzimide staining to detect morphological nuclear changes and/or by propidium iodide staining and flow-cytometry analysis to quantify sub-diploid apoptotic nuclei. The type of interaction between AT-101 and radiation was evaluated by calculating the Combination Index (CI) and by performing isobolographic analysis. For the pharmacokinetic analysis, plasma AT-101 levels were measured by HPLC in blood samples collected from patients enrolled in our clinical phase I/II study. These patients with locally advanced HNSCC were treated with standard cisplatin-based chemoradiotherapy and received dose-escalating oral AT-101 in a 2-weeks daily schedule every 3 weeks.

**Results:**

*In vitro* results showed that AT-101 enhances radiation-induced apoptosis with CI’s below 1.0, indicating synergy. This effect was sequence-dependent. Clonogenic survival assays demonstrated a radiosensitizing effect with a DEF_37_ of 1.3 at sub-apoptotic concentrations of AT-101. Pharmacokinetic analysis of patient blood samples taken between 30 min and 24 h after intake of AT-101 showed a dose-dependent increase in plasma concentration with peak levels up to 300–700 ng/ml between 1.5 and 2.5 h after intake.

**Conclusion:**

AT-101 is a competent enhancer of radiation-induced apoptosis in HNSCC *in vitro*. In addition, *in vitro* radiosensitization was observed at clinically attainable plasma levels. These finding support further evaluation of the combination of AT-101 with radiation in Bcl-2-overexpressing tumors.

## Background

The current standard treatment for advanced head and neck squamous cell cancer (HNSCC) is concurrent platinum-based chemoradiotherapy [1]. Despite encouraging results, treatment is still associated with significant toxicity and too many locoregional recurrences [2]. Besides dose-escalation strategies, molecular targeted drugs represent a new and promising approach to further improve treatment results [3]. HNSCC is frequently characterized by high expression levels of Bcl-2 family members, in particular anti-apoptotic Bcl-2 and Bcl-xL_,_ which has been associated with radio- and chemoresistance and poor clinical outcome [4–8].

Bcl-2 family proteins are key regulators of apoptotic pathways [9]. The family consists of the pro-apoptotic Bcl-2 Homology 3 (BH3) domain-only proteins, effector proteins Bax, Bak, and the pro-survival proteins Bcl-2, Bcl-xL, Bcl-w, Bfl-1, Mcl-1 and Bcl-B. Bax and Bak, upon their activation, induce mitochondrial membrane permeabilization by forming large homomultimeric pores. The activity of Bax and Bak is counteracted by the pro-survival Bcl-2 proteins that prevent their homomultimerization. In response to apoptotic stimuli, BH3-only proteins (Bid, Bim, Bad, Puma and Noxa) activate Bax and Bak by direct interaction, by releasing activated Bax and Bak from their pro-survival counterparts, or more indirectly, by liberating other BH3-only proteins from pro-survival Bcl-2 proteins, allowing these to activate Bax and Bak. BH3-mimetics represent a novel class of selective anti-cancer drugs that mimic the function of BH3-only proteins to induce tumor cell kill, and an appealing strategy to overcome resistance to anti-cancer therapies [10].

Gossypol was one of the first natural BH3-mimetics and has been identified as a potent inhibitor of Bcl-xL and Bcl-2 [11]. It is a polyphenolic dialdehyde derived from natural cottonseed and was originally applied as an anti-fertility agent [12]. Gossypol induces apoptosis in tumor cells with high levels of Bcl-xL and/or Bcl-2 expression, while leaving normal cells with low expression (such as fibroblasts and keratinocytes) relatively unaffected [13, 14]. Racemic (±)-gossypol consists of 2 enantiomers: (+)-gossypol and (-)-gossypol. (-)-Gossypol, also indicated as and from here on denoted AT-101, binds with high affinity to Bcl-xL, Bcl-2 and Mcl-1, and is more potent in inducing apoptosis compared to (+)-gossypol [11, 13, 15, 16].

Modulation of apoptosis is a promising strategy to improve radiation-induced tumor cell kill [3]. We demonstrated in a previous study in leukemic cell lines that the combination of radiation and AT-101 induced more apoptosis than the summation of their separate effects [16]. This combined effect was additive to synergistic, consistent with results generated in other tumor cell models [17, 18].

Clinical trials have demonstrated that AT-101 is well tolerated as a single agent [19–21] and in combination with other conventional therapies, including docetaxel/prednisone and cisplatin/etoposide [22, 23]. In our current phase I/II clinical study, we evaluate the feasibility, toxicity profile and pharmacokinetics of AT-101 in combination with cisplatin-based chemoradiotherapy in patients with locally advanced HNSCC.

The present report describes results from *in vitro* studies on the interaction between AT-101 and radiation in HNSCC cell lines, and from the pharmacokinetic analyses of our clinical phase I/II study in HNSCC patients. We showed that AT-101 is a potent enhancer of radiation-induced apoptosis *in vitro*, and importantly, that *in vitro* radiosensitization was observed at clinically achievable plasma levels.

## Materials and methods

### Reagents

(-)-Gossypol/AT-101 was provided by Ascenta Therapeutics, Inc. (San Diego, CA, USA). Stock solutions were prepared in dimethylsulfoxide to a concentration of 20 mM and stored at 4 °C. Prior to use an aliquot was diluted in Dulbecco’s modified Eagle’s medium (DMEM; GIBCO-BRL, Paisley, Scotland). Polyclonal rabbit anti-Bcl-xL and anti-Mcl-1 was from Cell Signaling Technology, and monoclonal mouse anti-Bcl-2 from Sigma-Aldrich.

### Cell culture

Two human head and neck squamous cell carcinoma (HNSCC) cell lines were used in this study. UM-SCC-11B was derived from a primary tumor of the larynx. This cell line was established at the laboratory of Dr. T.E. Carey (University of Michigan, Ann Arbor, MI, USA). VU-SCC-OE, an oral cavity carcinoma cell line, was a kind gift of Professor R.H. Brakenhoff (Department of Otolaryngology/Head and Neck Surgery, VU University Medical Center, Amsterdam, The Netherlands). These cell lines were grown in DMEM supplemented with 8 % heat-inactivated fetal calf serum, glutamine (2 mM), penicillin (50 U/ml) and streptomycin (50 μg/ml) in a humidified incubator with 5 % CO_2_ at 37 °C. These cell lines were tested to exclude *Mycoplasma* infection.

### Western blotting

To assess expression levels of Bcl-2, Bcl-xL, and Mcl-1, Western blot analysis was performed as previously described [16]. Equivalent protein loading was confirmed by total protein staining with 0.4 % Ponceau Red in 3 % trichloroacetic acid for 5 min. In these experiments blots were probed with Bcl-xL polyclonal antibody (1:1000) in 5 % nonfat dry milk, Bcl-2 monoclonal antibody (1:000) in 1 % nonfat dry milk, and Mcl-1 polyclonal antibody (1:1000) in 5 % BSA. After secondary horseradish peroxidase-conjugated antibody incubation, proteins were detected using the ECL detection system (GE Healthcare, Buckinghamshire, UK) and exposed to Amersham Hyperfilm MP (GE Healthcare, Buckinghamshire, UK).

### Irradiation procedure

For *in vitro* irradiation experiments, cells were exposed to gamma rays from a Gammacell® 40 Exactor (Best Theratronics Ltd. Ottawa, Ontario, Canada) at a dose rate of approximately 1 Gy/min. In control conditions, cells were sham-irradiated.

### Apoptosis assay

Apoptosis was assessed by staining with *bis*-benzimide to detect morphological nuclear changes or by propidium iodide staining and FACScan analysis to determine the percentage of subdiploid apoptotic nuclei as described earlier [16].

### Clonogenic survival assay

Cells were irradiated, 20 h later plated and allowed to attach for 6 h. AT-101 was then added and maintained in the culture medium for another 72 h. AT-101 was subsequently washed away and fresh medium was added. Cells were cultured for at least 14 days to allow colony formation. Colonies were fixed and stained with 0.2 % crystal violet/2.5 % glutaraldehyde. Colonies consisting of 50 cells or more were counted. The surviving fraction of cells was calculated by normalizing plating efficiency values of the treated samples to the untreated controls. Dose enhancement factor was determined at surviving fraction of 0.37 (DEF_37_).

### Statistical analysis

To characterize the interaction between ionizing radiation and AT-101 the Combination Index (CI) was calculated and isobolographic analysis was performed. The CI was calculated according to the classic isobologram equation described by Chou and Talalay [24]: CI = (D)_1_/(D_x_)_1_ + (D)_2_/(D_x_)_2_

In this equation, (D_x_)_1_ and (D_x_)_2_ represent the doses D_x_ of compounds 1 and 2 alone required to produce an effect, and (D)_1_ and (D)_2_ represent isoeffective doses D when compounds 1 and 2 are given simultaneously. The Combination Index can either indicate additivity (CI = 1), synergism (CI < 1) or antagonism (CI > 1). For isobolographic analysis, dose response curves of both AT-101 and radiation were generated using Graph Pad Prism 4.0 software. From each combination effect classic isobolograms were constructed [25]. A combination point below or above the envelope of additivity indicated a synergistic or antagonistic interaction between both stimuli, respectively.

### Clinical phase I/II trial

#### Patient selection criteria

Patients were eligible when aged 18 years or older, with stage III or IV, M0 histologically proven locally advanced inoperable HNSCC of the oral cavity, oropharynx or hypopharynx, and performance status WHO 0–2. Patients had no prior radiotherapy to the head and neck region or prior cisplatin-based chemotherapeutic treatment. Patients were required to have adequate hematologic, liver and renal function, and no uncontrolled arrhythmia. The study was approved by the Ethical Review Committee of The Netherlands Cancer Institute. Signed written informed consent was required before study entry.

#### Study design

Patients received standard cisplatin-based chemoradiotherapy (consisting of 70 Gy delivered in 35 fractions over 7 weeks, concurrently with 3-weekly 100 mg/m^2^ cisplatin i.v.) combined with dose-escalating oral administration of AT-101 in a 2-weeks daily schedule every 3 weeks. The starting dose of AT-101 was 10 mg daily and dose-escalation was in steps of 10 mg. Based on previously reported pharmacokinetic parameters [26] AT-101 was daily administered 2 h prior to fractionated radiation. The primary endpoint of this study was tolerability of AT-101 administration in combination with standard chemoradiotherapy. Secondary endpoints included pharmacokinetics of AT-101.

#### Pharmacokinetic evaluation

Blood samples were collected at 30 min after AT-101 intake, and after 1, 2, 3, 4, 5, 6, 7, 8 and 24 h. The 3 ml whole blood samples were collected in EDTA tubes and mixed with 0.3 ml 0.2 M freshly prepared reduced gluthatione, and centrifuged at 4 °C. After centrifugation the plasma was transferred in equal portions into 2 tubes containing 75 μl 25 mM acetonitrile maleic anhydride that was air-dried. The samples were stored at −80 °C until analysis. Plasma concentrations of AT-101 were determined by an HPLC-UV method derived from literature [26] which was optimized and validated. In short, an Agilent Zorbax Stable Bond C-18 column (150 x 4.6 mm I.D. 3.5 μm particle size) was used. Mobile phase consisted of 20 % 10 mM KH_2_PO_4_ : 80 % acetonitrile, at the flow rate of 1.0 ml/min. AT-101 was detected with a UV detector at 236 nm. Quantification was performed using calibration standards. An accelerated stability study was conducted at four different temperatures (37 °C, 21 °C, 4 °C and −20 °C) and led to a prediction of approximately 88.7 % of the original AT-101 concentration in the patient samples up to four years of storage at −80 °C.

## Results

### AT-101 target proteins are expressed in HNSCC cell lines

**Fig. 1:**
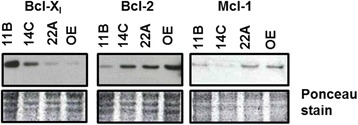
Expression of Bcl-xL, Bcl-2, and Mcl-1 in HNSCC. Western blot analysis demonstrating the expression of the anti-apoptotic proteins Bcl-xL, Bcl-2, and Mcl-1 in four different head and neck cancer cell lines, UM-SCC-11B, UM-SCC-14C, UM-SCC-22A and VU-SCC-OE

Western blot analysis demonstrated that HNSCC cell lines, specifically UM-SCC-11B, UM-SCC-14C, UM-SCC-22A and VU-SCC-OE, all expressed the anti-apoptotic proteins Bcl-xL, Bcl-2, and Mcl-1 (Fig. 1). Further investigation revealed that all four cell lines showed responsiveness to both radiation and AT-101 with ED_50_ values between 6 Gy and 16 Gy for radiation and ED_50_ values between 16 μM and 44 μM for AT-101. We continued our experiments with UM-SCC-11B and VU-SCC-OE since Bcl-xL and Bcl-2, the major targets of AT-101, were most prominently expressed in UM-SCC-11B and VU-SCC-OE, respectively. Mcl-1, a less predominant target of AT-101, was expressed at lower amounts.

### Radiation and AT-101 induce apoptosis in HNSCC cell lines

**Fig. 2:**
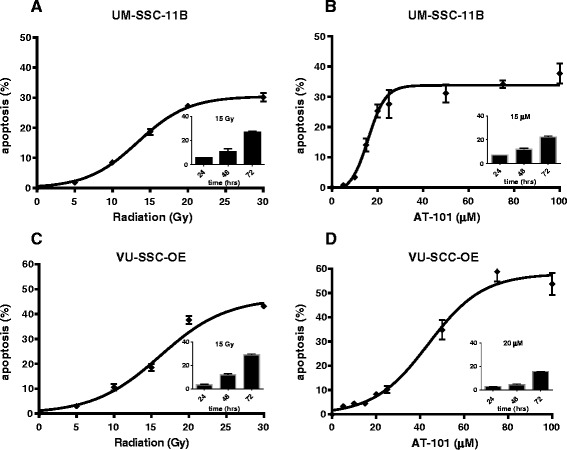
Dose- and time-dependent induction of apoptosis by radiation (**a**,**c**) and AT-101 (**b**,**d**) in HNSCC cell lines UM-SCC-11B (**a**,**b**) and VU-SCC-OE (**c**,**d**). Apoptosis was quantified 72 h after radiation and 48 h after exposure to AT-101. Data represent mean values ± SEM of an average of 11 independent experiments, performed in duplicate. Inserts show the time-dependency of radiation- and AT-101-induced apoptosis at the indicated doses

Radiation and AT-101 induced apoptosis in a time- and dose-dependent fashion (Fig. 2). UM-SCC-11B showed a steep dose–response curve up to 25 μM AT-101; in this cell line no further increase in apoptosis was detected up to 100 μM AT-101. Inserts show the time-dependency for both treatments. Apoptosis, induced by radiation and AT-101, as well as the combinations, results of which are described in the next paragraph, could be inhibited by pan-caspase inhibitor Z-VAD-FMK. In addition, we verified apoptosis induction by determining caspase 3 activation by methods described in [27] data not shown).

### Combined effects of radiation and AT-101 are synergistic

**Fig. 3:**
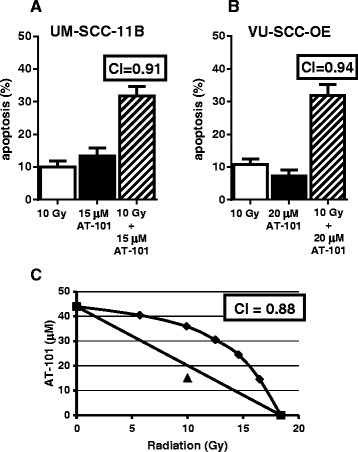
Synergistic interaction between radiation and AT-101. Figure 3**a** and **b** illustrate apoptosis induction in two HNSCC lines after radiation, AT-101 and their combination at the indicated doses. For the combination experiments, AT-101 was administered 24 h after radiation. These graphs represent the results of at least 3 independent experiments performed in duplo. Figure 3**c** shows an isobolographic analysis of the combined effect of 29 % apoptosis induced by 10 Gy radiation and 15 μM AT-101. The same level of apoptosis can be induced by 18.4 Gy of radiation or 44 μM of AT-101, respectively. The combination point (▲) below the envelope of additivity indicates a synergistic interaction between both stimuli. Note that in all conditions shown, the CI that refers to the combination index, is smaller than 1.0, indicating synergy as well

As demonstrated in Fig. 3a and b, the combination of radiation and AT-101 leads to a more than additive apoptotic effect. Isobolographic analysis, a statistical method to determine the type of interaction, shows a synergistic increase of apoptosis in VU-SCC-OE (Fig. 3c). Isobolographic analysis could not be performed in UM-SCC-11B, because the maximum levels of apoptosis induced by either radiation or AT-101 in these cells did not exceed the apoptosis levels after combined treatment, implicating synergy. Consistent with this observation the CI’s are below 1.0, in these cells.

### Synergistic interactions between radiation and AT-101 are sequence dependent

**Fig. 4:**
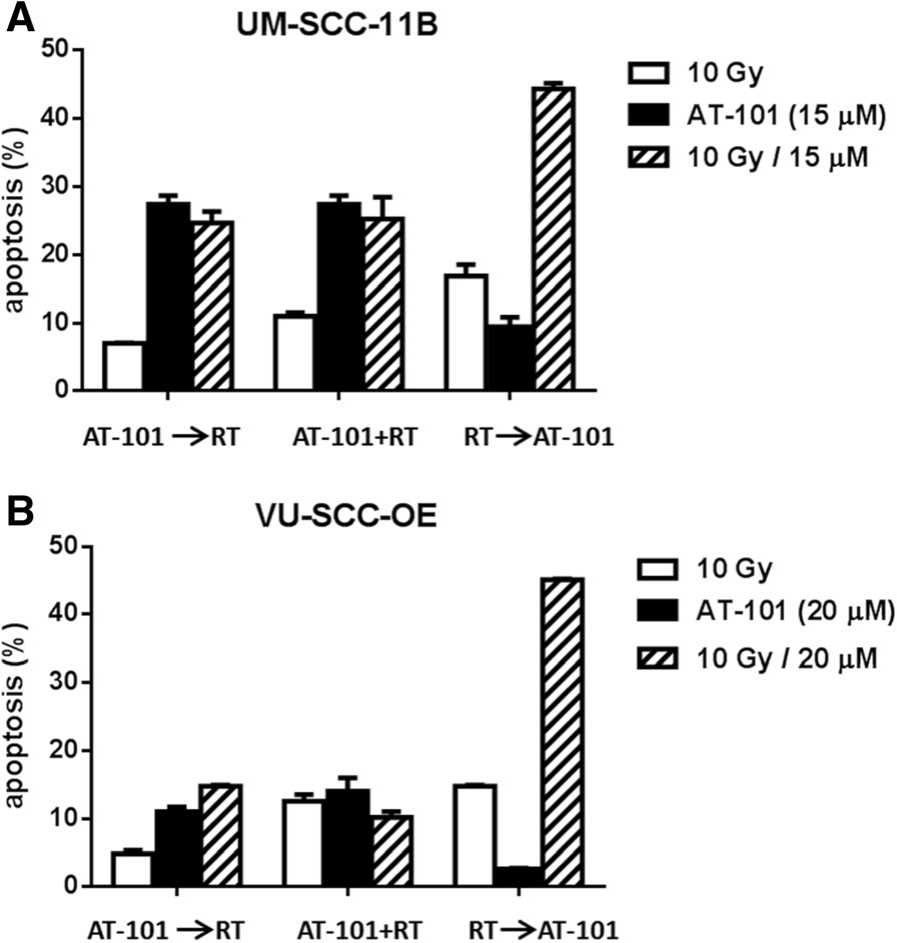
Sequence-dependent interaction between radiation and AT-101. Radiation and AT-101 were applied either concurrently or sequentially at the indicated doses. In the sequential schedule, AT-101 was administered either 24 h before radiation or 24 h after radiation.**a**: UM-SCC-11B; **b**: VU-SCC-OE. Data presented are representative of at least two experiments in both cell lines

Previously, it has been shown in other cell systems that the synergistic interaction between radiation and AT-101 can be sequence-dependent [16, 18]. We therefore also assessed this phenomenon in our HNSCC cell lines by comparing AT-101 administration 24 h before, during and 24 h after irradiation. Only when radiation preceded AT-101 treatment, this synergistic increase of apoptosis was found (Fig. 4).

### Clonogenic survival assays

**Fig. 5:**
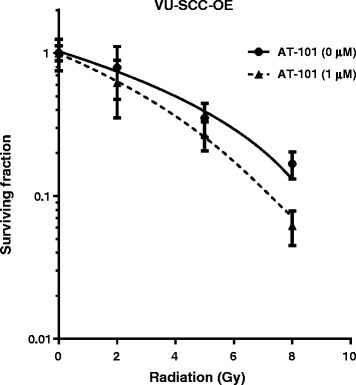
Effect of AT-101 on clonogenic survival after radiation. Clonogenic survival curves of VU-SCC-OE cells after radiation in the absence (solid line) or presence of 1 μM AT-101 (normalized dashed line) are shown. Graphs show representative curves of at least 3 experiments in triplicate, error bars represent SEM.

To determine the impact of AT-101 on long-term survival after radiation, we performed clonogenic survival assays. At concentrations of AT-101 below 2 μM (i.e. in the range where no significant apoptosis induction was found; Fig. 2) no significant decrease in clonogenic survival was observed (data not shown). Fig. 5 shows that AT-101 at a final concentration of 1 μM reduced clonogenic survival after radiation (DEF_37_ = 1.3), consistent with a radiosensitizing effect of AT-101.

### Pharmacokinetic analysis in patient samples

**Fig. 6:**
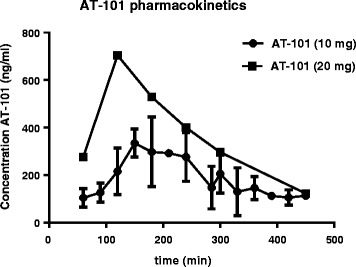
Clinical pharmacokinetics of AT-101. AT-101 concentration was measured by HPLC in patient plasma samples collected at 30 min, 1, 2, 3, 4, 5, 6, 7, 8 and 24 h after oral intake of AT-101. Pharmacokinetic profiles are shown for the AT-101 dose levels of 10 mg (*n* = 13) and 20 mg (*n* = 1). Data represent mean values ± SD

In an ongoing phase I/II trial, patient blood samples were collected between 30 min and 24 h after oral intake of AT-101. Two dose levels could be analyzed; 10 mg (*n* = 13) and 20 mg (*n* = 1). Fig. 6 shows the pharmacokinetic profiles with a dose-dependent increase in plasma concentration, peaking between 1.5 and 2.5 h at approximately 300 and 700 ng/ml for the 10 mg and 20 mg dose level, respectively. These levels correspond with 0.6–1.35 μM AT-101.

## Discussion

Despite significant improvements in the treatment of patients with inoperable head and neck cancer, recurrence rates remain unacceptably high. Thus, there is a clear need to develop new therapeutic approaches to further enhance the anti-tumor efficacy of existing standard regimens, such as cisplatin-based chemoradiotherapy. Overexpression of anti-apoptotic members of the Bcl-2 family is frequently observed in HNSCC and has been associated with resistance to radio- and chemotherapy and with poor prognosis [4–8]. Therefore, in the present studies we focused on AT-101, a BH3 mimetic and small molecule inhibitor of pro-survival Bcl-2 proteins, and its potential to increase the cytotoxic effect of radiation in HNSCC *in vitro*. Our results show that AT-101, only when added after radiation, enhances apoptosis to synergistic levels, and acts as a radiosensitizer in clonogenic survival assays. To address the question whether the effective *in vitro* concentrations of AT-101 correspond with those achievable in a clinical setting, we determined AT-101 plasma levels in a subset of patients included in our phase I/II trial. Indeed, plasma levels of AT-101 were comparable with the low micromolar radiosensitizing concentrations *in vitro*.

A synthetic class of BH3 mimetics that has been developed recently shows interesting results regarding their capacity to radiosensitize cancer cells, including ABT-737, a molecule with high affinity for Bcl-2 and Bcl-xL, and its analogue the clinically more favorable, ABT-263. These compounds do not or only weakly target Mcl-1 [28, 29], whereas AT-101 demonstrates a more favorable binding profile towards Mcl-1 [30]. Studies with ABT-737 and ABT-263 now suggest that Mcl-1 plays a role in resistance to these compounds [28, 31]. A recent study on radiation and BH3 mimetics in breast cancer showed treatment with ABT-737 alone elevated Mcl-1. ABT-737 together with radiation, however, demonstrated a synergistic effect on breast cancer cells by downregulation of Mcl-1 [32]. Also a study on pancreatic cancer cells evaluated Mcl-1 as a target for radiosensitization [33]. These studies suggest that ABT-737 or ABT-263 may be suboptimal to target Mcl-1, in particular in combination with radiation.

AT-101, the cis- or (-)-enantiomer of racemic Gossypol, is a naturally occurring polyphenolic dialdehyde derived from cottonseed. Gossypol enantiomers, including AT-101, have been used as cytotoxic agents *in vitro* and *in vivo* using different tumor cell lines from both solid [13–15, 34, 35] and leukemic origin [16]. Importantly, only minimal effects were observed on normal cells [13, 14], indicating a certain degree of tumor selectivity. Several groups have investigated the combined effects of AT-101 and chemo- or radiotherapy [17, 18, 36]. In human prostate cancer cells, AT-101 potently enhanced radiation-induced apoptosis and growth inhibition and reduced clonogenic survival [18]. We showed in two human leukemic cell lines an additive to synergistic interaction between radiation and AT-101 [16]. Interestingly, HNSCC cell lines made resistant to cisplatin retained their apoptosis sensitivity towards AT-101 [13, 34]. *In vivo*, the anti-tumor effect of AT-101 has been tested as single agent [37] and in combination with radiation [18] and chemotherapy [38]. In an orthotopic xenograft model of HNSCC with high Bcl-xL expression, daily i.p. injection of AT-101 resulted in a significant tumor growth delay as compared to control animals [37]. Histopathological analysis showed a decrease in mitotic index and an increase in apoptosis in the AT-101-treated tumors. Treatment was well tolerated, as reversible moderate weight loss was the only observed side effect. In a prostate cancer xenograft model daily oral AT-101 was compared with fractionated radiotherapy and with the combination of both [18]. Especially when larger tumors were treated, only the combination of AT-101 and radiation achieved significant anti-tumor activity. Tumor tissue specimens showed not only a significant increase in apoptosis after combined treatment, but also a strong inhibition of tumor angiogenesis. No significant weight loss or obvious organ toxicities were observed. From these experimental studies it can be concluded that AT-101 has significant anti-cancer activity as single agent, but is much more effective in combination with other cytotoxic regimens like cisplatin and radiation therapy. Upon oral administration, it has demonstrated little toxic side effects in animals.

Clinical studies on AT-101 as single agent or combined with chemotherapy are limited, but indicate good tolerability [19–23]. Clinical experience with AT-101 in combination with (chemo-)radiotherapy is even sparser [39], but accumulating from several ongoing or recently completed phase I studies, including ours in HNSCC.

In the present studies, we demonstrate a dose- and time-dependent increase in apoptosis by radiation and AT-101 in two human HNSCC cell lines expressing the pro-survival Bcl-2 family members Bcl-xL, Bcl-2, and Mcl-1, all established targets of AT-101. By performing isobolographic analysis and calculating the Combination Indices, we characterized the type of interaction between both treatments as synergistic. These findings are in agreement with the results from studies using other cell systems [16, 18]. We also found that this synergistic interaction between radiation and AT-101 was only present when AT-101 was added after radiation, as observed in other cell systems as well [16, 18]. This apparent sequence-dependency is poorly understood and thought to be cell cycle related [18]. In a previous study [16], we provided evidence that activation of the SAPK/JNK signal transduction pathway plays a significant role in AT-101-induced apoptosis. Because radiation is a well-known activator of SAPK/JNK [40] and it has been shown that SAPK/JNK translocates to the mitochondria upon irradiation where it phosphorylates and inactivates Bcl-xL, Bcl-2 and Mcl-1 [41–43], this mechanism may provide an alternative explanation for the observed sequence-dependency.

It has been shown that genetic or pharmacological modulation of radiation-induced apoptosis frequently also impacts on radiosensitivity [44, 45]. Therefore, we evaluated the effect of AT-101 on clonogenic survival after irradiation. Indeed, at concentrations that do not induce significant levels of apoptosis, a clear radiosensitizing effect was observed. This radiosensitizing potential of AT-101 most likely depends on the cell type studied, as it has been demonstrated in certain cell systems [17, 36], but not in others [16]. In a number of different tumor cell lines, including HNSCC, radiosensitization by AT-101 was found to result from reduced double-strand break repair [46]. Others have suggested that increased autophagic cell death plays an important role in AT-101-induced inhibition of clonogenic survival of irradiated glioblastoma cells [36].

To determine whether the radiosensitizing concentrations of AT-101 are comparable with the plasma levels that can be achieved in patients, we analyzed the pharmacokinetic data collected in our clinical phase I/II study. At daily doses of 10–20 mg, plasma levels peaked around 2 h after intake, suggesting slow absorption. Maximum plasma concentrations were in the micromolar range, corresponding to those that induced radiosensitization *in vitro*. Both the maximum observed plasma concentration and the time to reach this value are similar to other reports [37]. Although it is difficult to compare *in vitro* with *in vivo* drug concentrations, it is reassuring that no major differences were found. Regarding the scheduling of radiotherapy and AT-101, daily radiation was given just prior to or at maximal plasma concentrations. Evidently, more clinical studies are needed to define safety and efficacy of AT-101 in combination with radiation, and to determine intra-tumoral drug concentrations for optimal scheduling.

## Conclusions

In summary, we showed that AT-101 synergistically enhanced radiation-induced apoptosis in HNSCC *in vitro* in a sequence-dependent manner. In addition, *in vitro* radiosensitization was observed at clinically achievable plasma levels. These findings provide a rationale to further evaluate AT-101 in combination with standard (chemo-)radiation in Bcl-2-overexpressing tumors, such as head and neck squamous cell carcinoma.
